# An Affordable Fabrication of a Zeolite-Based Capacitor for Gas Sensing

**DOI:** 10.3390/s20072143

**Published:** 2020-04-10

**Authors:** Salvatore Andrea Pullano, Francesco Falcone, Davide C. Critello, Maria Giovanna Bianco, Michele Menniti, Antonino S. Fiorillo

**Affiliations:** Department of Health Sciences, University “Magna Græcia” of Catanzaro, Viale Europa, 88100 Catanzaro, Italy; cesco.falcone@gmail.com (F.F.); critello@unicz.it (D.C.C.); mg.bianco@unicz.it (M.G.B.); menniti@unicz.it (M.M.)

**Keywords:** zeolite, adsorption process, nanoporous materials, sensor application

## Abstract

The development of even more compact, inexpensive, and highly sensitive gas sensors is widespread, even though their performances are still limited and technological improvements are in continuous evolution. Zeolite is a class of material which has received particular attention in different applications due to its interesting adsorption/desorption capabilities. The behavior of a zeolite 4A modified capacitor has been investigated for the adsorption of nitrogen (N_2_), nitric oxide (NO) and 1,1-Difluoroethane (C_2_H_4_F_2_), which are of interest in the field of chemical, biological, radiological, and nuclear threats. Sample measurements were carried out in different environmental conditions, and the variation of the sensor electric capacitance was investigated. The dielectric properties were influenced by the type and concentration of gas species in the environment. Higher changes in capacitance were shown during the adsorption of dry air (+4.2%) and fluorinated gas (+7.3%), while lower dielectric variations were found upon exposure to N_2_ (−0.4%) and NO (−0.5%). The proposed approach pointed-out that a simple fabrication process may provide a convenient and affordable fabrication of reusable capacitive gas sensor.

## 1. Introduction

The adsorption/desorption of a gas molecule onto a surface, and its detection, have deserved extensive research in the last decades since the increasing interest in the monitoring of hazardous species (chemical, biological, radiological, nuclear (CBRN) threats; environmental; and health-related) [1,2,3,4,5]. Even more sophisticated laboratory equipment, such as gas chromatography and mass spectrometry, are widespread at laboratory level, achieving the desired detection performances with a very limited portability [6]. However, continuous efforts are devoted to the development of compact and low-impact sensors for volatile chemical species with improved sensitivity, specificity, accuracy, response, and recovery times [7]. To this end, a plethora of transduction phenomena (chemiresistive, capacitive, mass-sensitive, calorimetric, and optical) were investigated to propose various technological solutions for the analysis of complex matrices [8,9,10,11,12,13]. From the analytical point of view, different models were developed to propose a relationship among the number of adsorbed/desorbed molecules, their interactions, and the sensor response, such as the Langmuir and Wolkenstein’s models [14,15,16]. However, the development of an ideal gas sensor is extremely difficult, and the lack of selectivity remains the major limitation. Therefore, the development of arrays of non-selective sensors, together with a dedicated signal analysis, was amply investigated to improve the overall performances, often in accordance with bio-inspired techniques (electronic nose) [11,17].

Among the materials used in the field of chemo- and bio-sensing, porous materials with their unique filtering characteristics, and the possibility of tailoring the morphology and the size of the pores, allow the design of dedicated gas sensors. In particular, metal oxides and silicon with porous and nanosheets structure (large surface to volume ratio and related quick diffusion of molecules), were amply investigated for the detection of low-trace gases [12,13,14,15,16,17,18,19,20,21,22]. Another class of interesting porous materials is represented by zeolites, recently named as one of the breakthrough research topics [23]. Their chemical structure confers to the material interesting catalytic properties, ion exchange, and geometric selectivity, which make them particularly useful as molecular sieves against specific molecules [24,25,26,27,28,29]. The adsorption process is closely related to the affinity of the zeolite framework to polar or polarizable molecules and to the kinetic diameter. Recent investigations have highlighted a molecular “trapdoor” mechanism in the chabazite-type zeolite, in which, based on the position of extra framework cations, a doorway for gas molecules access can be allowed or blocked [30,31]. The adsorption is driven by a critical gas admission temperature, which is different from gas to gas. The interesting molecular-dependent conduction mechanism results promising in the use of zeolite as selective sensing materials in next generation electrical-based gas sensors [31,32]. From the electrical point of view, zeolite framework contains both conductive and insulating regions [30]. The electrical conductivity and dielectric relaxation of zeolite evidenced that it is mainly due to the migration of cations (typically alkali metal or alkaline earth metal cations).

We have recently investigated a deposition technique based on the spin-coating of a mixture of zeolite with high iodine value oil followed by a low temperature annealing process [32]. By this technique, it is possible to deposit films of various thicknesses (down to few microns). Hereafter, a capacitive zeolite-based gas sensor, which exploits the adsorption/desorption capabilities of zeolite, together with a simple and affordable fabrication technique is presented. 

The electrical characterization of an electric capacitor fabricated with zeolite 4A are presented. Specifically, the electric capacitance related to the adsorption of several gases (i.e., N_2_, NO, and C_2_H_4_F_2_) at isotherm condition and variable pressure was investigated. The proposed sensor exploits a simple fabrication process for a capacitive sensor manufacturing based on spin-coating technique. The results demonstrate that such approach may provide convenient and affordable means for gas sensor manufacturing.

## 2. Materials and Methods

### 2.1. Electrical Conductivity of Zeolite

Zeolites are a family of natural or synthetic crystalline materials with interconnected aluminosilicate building blocks [24]. The silicon and aluminum atoms are connected to each other through shared oxygen atoms, therefore giving well-defined 3D frameworks and porosity [17]. Zeolites include a variety of crystalline materials with regular intra-crystalline pores and channels having molecular dimensions that can range from 3 to 12.12 Å, with interesting ion-exchange, molecule-trapping, and sieving capabilities [24,25]. Negatively charged building blocks are balanced by cations, electrostatically bound to the framework and mobile along the channels. In recent years, electrodes characterized by a zeolite-based composite layer have been developed [33]. Most of the zeolites’ properties are very promising for the fabrication of gas sensors; in particular, they can be assimilated to conductive gas sensors. It is therefore possible to exploit the above-mentioned properties to fabricate a nanoporous matrix gas sensor [34,35,36,37,38]. Physical adsorption is mainly caused by the van der Waals forces and electrostatic interactions between the adsorbate molecules and the atoms of the adsorbent surface; since the attraction forces are weak, the adsorption process can be easily reversed by heating or decreasing the environmental pressure. 

**Figure 1:**
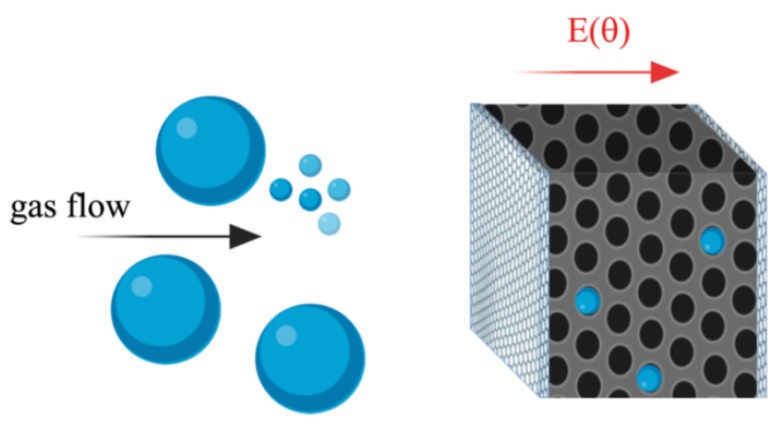
Proof of concept of the zeolite-based gas sensor. A nanoporous layer acts as a molecules-sensitive dielectric, allowing a capacitive detection of the analyte, which results in an electrical signal correlated to the gas species adsorbed. The metal layers, together with the nanoporous dielectric, form a parallel plate capacitor whose electrodes are permeable to the gas stream, in order to allow a full diffusion of the molecules inside the zeolite. E(θ) represents the electric field between the two large parallel plates which is expected to be function of θ, defined as the number of adsorbed molecules compared to the total number of adsorption sites.

Each type of zeolite possesses a specific electrical conductivity. Zeolites’ conduction mechanisms involve the analysis of charges migration as a consequence of applied electric field. The latter creates a re-arrangement of mobile cations inside the zeolite framework, caused by metal electrodes, which can start a redox process, modulating an overall intrazeolitic electric current [39]. As the channel size and the cations dimensions approach each other, cations migration (conduction) is reduced, as in the case of zeolite A [40]. From the electric point of view, the nanoporous composite layer can be modeled as a set of conductive percolative paths (complete and incomplete) and dielectric regions (aluminosilicate framework). The literature has evidenced that, even though the low frequency permittivity of zeolite powder is typically low, the presence of other species or guest molecules (e.g., water) resulted in a variation of the overall permittivity [30,39]. Moreover, the combination of amorphous materials (vegetable oil) and zeolite grains resulted in a film with a higher structural integrity. According to Figure 1, the composite layer is expected to have an overall capacitive behavior (losses capacitor), influenced by the adsorption of gas molecules, which will affect the cations mobility and thus the AC conductivity. Moreover, apart from the changes in electric capacitance due to the gas interaction, size, and shape selectivity can be imparted to the sensitive layer [30,31,32]. Finally, the control of Si/Al ratio of the zeolite framework confers hydrophobic/hydrophilic properties to the materials, which make these solids specific for the adsorption in the gas or liquid phase with controlled sensitivity [41].

Zeolite Linde Type A (LTA), specifically Zeolite 4A powder was provided by Luoyang Jianlong Micro-nano New Materials Co., Ltd. (Yanshi, China). It is the sodium form of type A, contains spherical cavities with nominal diameter pore of 4.2 Å, and the chemical formula is given by

(1) $$Na_{86}^{+}\left[{\left(SiO_{2}\right)}_{106}\cdot {\left(AlO_{2}^{-}\right)}_{86}\right]\cdot H_{2}O$$

Sodium zeolite consists of a central cavity of 11.4 Å in diameter, eight-ring openings of 4.1 Å in diameter, and a void volume fraction of 47% [42]. The average grain size was evaluated using a Zetasizer Nano ZS (Malvern Instruments Ltd., Worcestershire, UK). Zeolite 4A powder is characterized by an average grain size of 450 nm, a bulk density ≥0.70 g/mL, a pH ≤11.00, and a static water adsorption ≥21.5 %wt. Refined food-grade soybean oil was used to prepare a mixture with zeolite LTA. The composition of the refined oil was 9,12-octadecadienoic acid (55.4%), 9-octadecenoic acid (26.3%), hexadecanoic acid (12.8%), octadecanoic acid (4.2%), and 12-octadecenoic acid (1.3%). A coverslip of 24 × 24 mm^2^ with a thickness of 100 µm (Knittel Gläser, Bielefeld, Germany) was used as substrate. The dry air (78.0% of N_2_, 20.9% of O_2_, 0.9% of Ar, 0.1% CO_2_, besides other gases in traces) and N_2_ (>99.99%) were obtained from ICOA (Vibo Valentia, Italy) while NO (>99.9%) and C_2_H_4_F_2_ (99.9%) were provided by Rivoira (Milano, Italy). Main gas characteristics are reported in Table 1.

### 2.2. Sensor Manufacturing and Characterization

**Figure 2:**
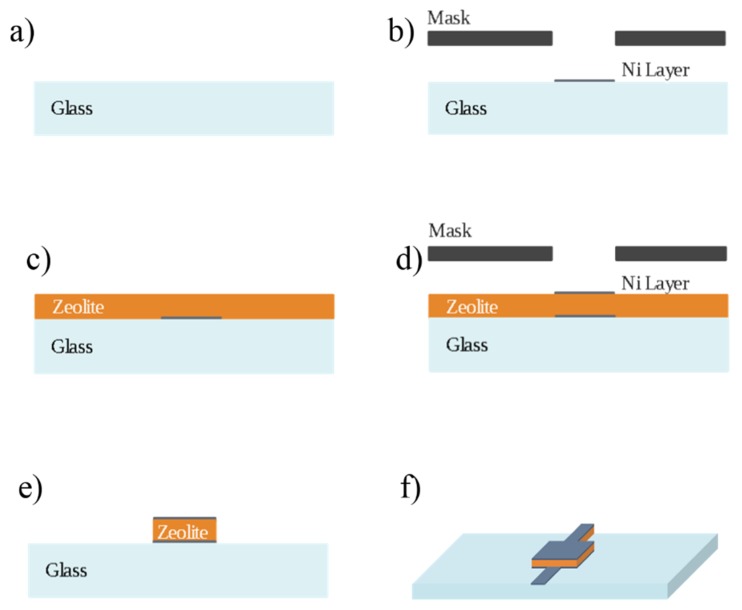
Fabrication procedure of gas sensitive nanoporous capacitor. (**a**) Starting from a cleaned glass substrate, (**b**) a thin Nickel electrode is deposited, and then (**c**) a zeolite composite layer is spun coated. (**d**) An upper Nickel electrode is deposited and, (**e**) a wet etching process is used for removing the zeolite excess, obtaining (**f**) a planar nanoporous capacitor.

A squared electrode with a 5-mm side was designed and manufactured (Figure 2a). A glass substrate with a thickness of 100 µm was used while a stencil mask was 3D printed for the patterning of metal electrodes. A first layer of Cr (thickness of 5 nm) and second layer of Ni (thickness of 100 nm) were firstly deposited on the glass coverslip. All the metal layers were deposited through e-beam physical vapor deposition (Varian technologies, Palo Alto, CA, USA) at a pressure of 5 × 10^−6^ Pa (Figure 2b). The electrode thus obtained was coated with a mixture of zeolite 4A and a high iodine value vegetable oil. The mixture of 70% of soybean oil and 30% of zeolite 4A w/w was mixed for 30 min using an Ultra-Turrax homogenizer at 25,000 rpm and kept in a sonicator for 20 min. Then, it was deposited on the metal-coated glass support by a Spin-Coater at 4000 rpm for 60 s (Figure 2c). Finally, the sample was annealed on a hot plate at 120° for 6 h. The annealing process allows the deposition of a nanoporous layer consisting of the zeolite grains, held together by a supporting matrix originated by oil degradation. The oil was chosen according to the number of double carbon bonds (the more the better) or, equivalently, a higher iodine value. A stencil mask was then used to deposit a 100-nm Ni upper metal electrode (Figure 2d), and a wet etching was then used to remove the zeolite in excess. Buffered hydrofluoric acid (BOE) 7:1 (HF: NH_4_F = 12.5:87.5%) in VLSI-quality was used to isotropically etch the zeolite composite layer (Figure 2e). The etched substrate was then rinsed with deionized water and dried under nitrogen flow. Finally, another annealing step was performed at 120 °C for 1 h.

**Figure 3:**
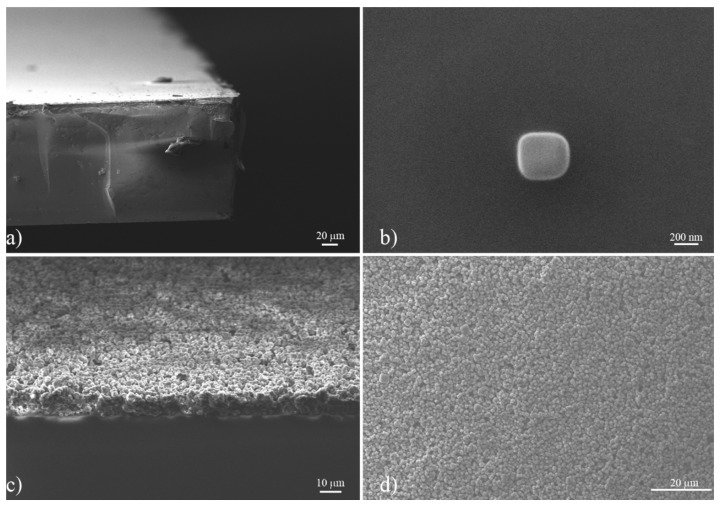
SEM images of: (**a**) the glass substrate; (**b**) the nanoporous grain composing the mixture; (**c**) the deposited composite layer after annealing process; and (**d**) the upper view of the layer.

As a result of a second metal electrode deposition, a contactable parallel face capacitor was fabricated (Figure 2f), in which the nanoporous layer acts as a dielectric. The morphology of the capacitor performed by Scanning Electron Microscope (EVO HD 15 Carl Zeiss) is reported in Figure 3. As evidenced, the glass metallized substrate (Figure 3a) was covered by a nanoporous layer, which appears as an agglomeration of cubical-shaped grains with an average size of 450 nm (Figure 3b,c). The composite layer is held together by a carbonic framework, resulting from the decomposition of the vegetable oil during the annealing process, with a strong adhesion on the substrate.

**Figure 4:**
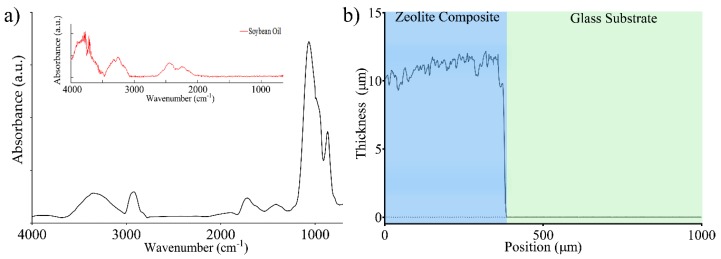
(**a**) FTIR Analysis of the zeolite composite layer, with the soybean oil spectrum in the inset. (**b**) Profilometric analysis of a sample of 10 μm in thickness.

In our recent studies, a nanoporous layer fabricated through spin-coating technique was investigated through Fourier Transform Infrared (FT-IR) spectroscopy and SEM microanalysis, evidencing that no other contaminants besides those introduced by zeolite and thermal degradation of the oil are present [3,11,33]. One of the prerequisites for zeolite gas adsorption is the permeation of gas molecules through the Ni layer, whose mechanism is widely investigated in [44,45]. FT-IR spectroscopy (Figure 4a) was performed on the nanoporous layer (without upper metal electrode) to characterize the zeolite layer. 

As previously reported in the literature, the spectra showed an area of higher transmittance between 800 and 1200 cm^−1^, which is due to the stretching of Si-O, Al-O and Al-OH bonds. The broad band at 3000–3600 cm^−1^ is due to the water content still present in the layer. The influence of oil, which usually lays in the band between 2100 and 2600 cm^−1^, was not observed after thermal annealing. Moreover, the peaks in the bands at 3000–2800 cm^−1^ and 1800–1600 cm^−1^ can be attributed to C-H stretching and carbonyl groups. While thermal treatment at 120 °C does not affect zeolite stability, it results in oil degradation (a reduction of C=C stretching and the secondary products), creating a thin adsorbing layer of zeolite grains adhered to the metallized glass substrate [46]. Adsorption characteristics of the layer were investigated by gravimetric analysis of H_2_O. A non-metallized sample was kept at 20 mbar overnight, then exposed to air with a relative humidity (RH) of 70%, and finally placed at 20 mbar after an annealing process. According to the gravimetric analysis, the layer evidenced variations of about 15 %wt, which are consistent with the data provided by the zeolite supplier, confirming the maintained adsorption capability of the layer. Morphological analysis performed through a Dektak 6M profilometer (Veeco, Plainview, NY, USA) evidenced an average layer thickness of the layer of 10.90 μm and a roughness R_q_ equal to 0.98 μm (Figure 4b). While thickness mainly influences the static capacitance C_0_, roughness is mainly due to the granularity of the mixture and the deposition process. Since the adsorption of gas molecules at room temperature was expected to influence the permittivity of the nanoporous composite through a purely adsorptive mechanism, C_0_ was evaluated at 20 mbar in anhydrous environment leading to 0.25 ± 0.001 nF @ 1 kHz. According to the geometry of the capacitor, the estimated relative permittivity (*ε_r_*) of the layer was 12.3 @ 1 kHz [47]. 

### 2.3. Measurement Procedure

Supposing negligible changes of the form factor *A/d* in the detection of gas molecules, the parallel plate capacitor formula is *C = ε*_0_*ε_r_A/d* (with *ε*_0_ the vacuum permittivity, *ε_r_* the relative permittivity, *A* the electrode area, and *d* the thickness of the dielectric layer). The latter involves changes in the permittivity due to the adsorption inside the porous structure of the capacitive sensor. 

**Figure 5:**
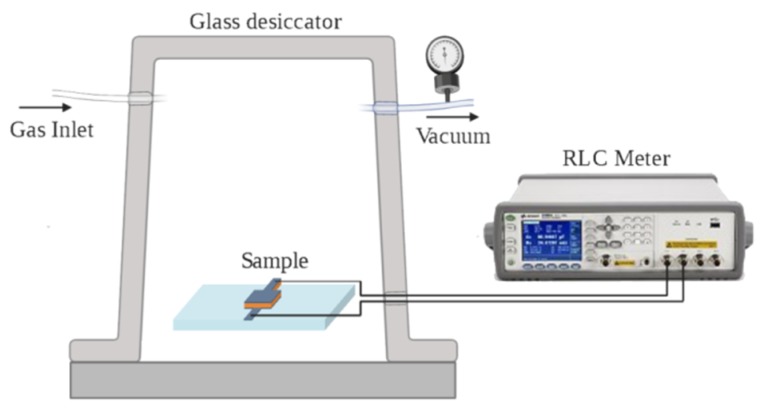
Setup used for the characterization of the zeolite-based capacitor for gas sensing (not to scale).

Before electrical characterization, electrical contacts were provided through silver-loaded conductive epoxy 8331-B (MG Chemicals^®^, Surrey, Canada). Characterization was performed by a static system, consisting of a vacuum bell jar, in which the sample, a temperature sensor, and all the electrical connections were placed (Figure 5). The sensor operated at controlled temperature (i.e., 23 °C), while parallel electrical capacitance was continuously evaluated [48]. The sample was placed inside a controlled vacuum bell jar at a distance of 0.2 m from the gas inlet/purge valve, and the electrical contacts to separate the gas flow from the sensor. A vacuum pressure gauge (TS-Z50A 0÷30 inHg, 0÷1 bar) was used to monitor the pressure inside the chamber. A mechanical pump maintained vacuum inside the chamber. Measurements were performed by a Keysight E4980AL RLC meter in the range from 150 Hz to 1 kHz, in order to analyze the changes in the dielectric composition of nanoporous electric capacitor due to the adsorption of the gas molecules in the environment. Initially, the vacuum bell jar was placed at 20 mbar, then the gas was injected, and electric capacitance measurement was performed at both constant pressure (time evolution) and variable pressure. The sensor was exposed to subsequent static gas concentration in the range 100–500 ppm in dry air. All the results reported are related to the adsorption of a single gas and no competitive adsorption phenomena were considered. After each set of measurements, the gas was leaked out, and the sensor was recovered by reducing the pressure at 20 mbar for 1 h.

## 3. Results and Discussion

**Figure 6:**
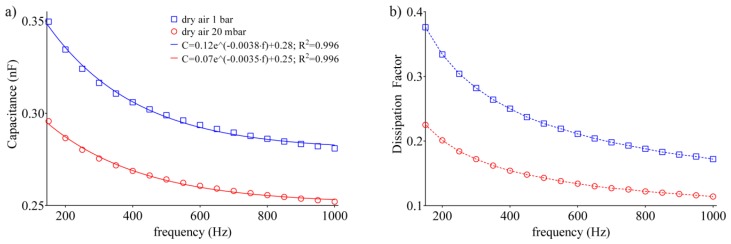
Relationship inside the vacuum chamber at 20 mbar and 1 bar dry air saturated environment of: (**a**) electric capacitance vs. frequency; and (**b**) dissipation factor vs. frequency.

The adsorption of gas species inside the zeolite cavities takes place both with physical (Van der Waals attraction) and chemical (covalent bonds) mechanisms, and electrostatic attraction forces often come into play. The adsorption capability of zeolites depends on several factors, including the distribution and the number of cations in their porous structure, the Si/Al ratio, the pore size, the polarization, and the size of the adsorbed molecules, the presence of water and other gases, and the presence of carbonates on their surface. Even experimental conditions, such as pressure and temperature, are factors that influence the adsorption capability of zeolites. Furthermore, the zeolite behaves as a Brønsted–Lowry acid or base, with the ability to exchange H^+^ ions. To evaluate the adsorption characteristics of the nanoporous capacitor, the polarizability of the material used as dielectric with a permittivity ε(ω), was investigated. Electric capacitance and dissipation factor (*DF*) were evaluated through a parallel-equivalent circuit model. In this case, dry air was used to preliminarily test the adsorption capability of the sensor. Prior to the analysis, the sample was maintained for overnight at 20 mbar to allow a fully desorption of the gas molecules previously adsorbed into the sample. A preliminary standard gravimetric analysis was performed to verify the adsorption capability of the deposited layer. Figure 6a,b reports the capacitance and the dissipation factor at a pressure of 20 mbar and 1 bar in the frequency range 150–1000 Hz. 

**Figure 7:**
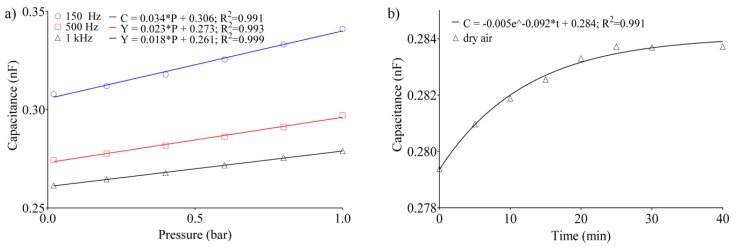
(**a**) Electric capacitance vs. pressure at three different frequencies onto the same sample. (**b**) Time evolution of the capacitance at constant pressure and temperature.

Results can be fitted with a one phase decay (R^2^ > 0.99), evidencing the characteristic decrease in capacitance (i.e., permittivity) vs. frequency of the type A zeolite. The latter is consistent with the data reported in the literature, according to which, at lower frequency, real part of permittivity (ε’) decreases prominently with frequency [49]. Being supported by the mobile metal ions in the framework, conduction is of ionic nature, which involves the displacement of heavier charges compared to electronic conduction. Thus, the decrease in capacitance is due to the fact that, increasing the frequency, the mobile cations follow the electric field with a lag, contributing less to the permittivity. Therefore, the different trend reported in Figure 6 is related to the excess of gas molecules adsorbed inside the pores of the dielectric. As a first analysis, we considered that the pores of zeolite 4A have a diameter of about 4.2 Å, and the air components have the following kinetic rays: N_2_ = 3.64 Å, O_2_ = 3.46 Å, Ar = 3.4 Å, CO_2_ = 3.3 Å, and H_2_O in the form of steam, about 2.65 Å. It can be immediately noticed how the dimensions of each component are less than the pores’ width. Therefore, all the molecules satisfy the zeolite’s selectivity property. The negatively charged unit blocks of the crystalline structure of zeolite 4A are balanced by Na^+^ cations and water molecules. During the adsorption process, the water molecules present in the air in the form of vapor bind more easily to the active sites due to the kinetic radius being much smaller than that of the other components, and for the greater affinity of the H^+^. A more detailed analysis of electric capacitance vs. pressure is shown in Figure 7a, in which the dry air adsorption for three representative frequencies (i.e., 150, 500 and 1 kHz) was evaluated by increasing the pressure inside the chamber from 20 mbar up to 1 bar in 60 s. After each acquisition, the sample was maintained at 20 mbar for 1 h. 

The capacitance trend assumes a behavior that is similar to a linear isotherm adsorption model, in which the capacitance is linearly related to the gas pressure. In this case, considering that the fraction of gas adsorbed can be modeled as *θ = k_L_P/(1 + k_L_P)*, where *k_L_* is the Langmuir constant (which takes into account the affinity of adsorbent respect to adsorbate, and is temperature dependent) and *P* is the gas pressure, the trend shown in Figure 7a can be assimilated to a low-pressure adsorption isotherm in which (*1* + *1 + k_L_P*) can be neglected obtaining a linearity higher than 0.99. Figure 7a evidences the sensor’s sensitivity in the range 20 mbar to 1 bar varies from 10 to 34 pF/bar depending on the frequency used (150, 500, or 1000 Hz). The evolution of the adsorption process was monitored at regular intervals of 5 min starting from a pressure of 20 mbar at given frequency of 1 kHz, and subsequently subjected to an increase in pressure through a constant air flow (Figure 7b). The results evidence how the adsorption dynamics are characterized by a *τ* = 10.78 min and a plateau at 284 pF; therefore, it is plausible that after this time the cavities of the zeolite film have saturated and therefore the transducer cannot longer adsorb other molecules, or, alternatively, the adsorption and desorption rate are in equilibrium.

**Figure 8:**
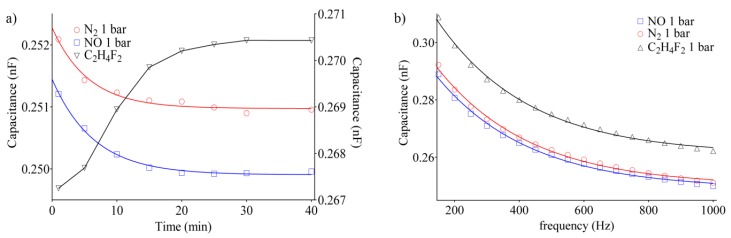
Adsorption of N_2_, NO, and C_2_H_4_F_2_ by the Zeolite-based sensor. (**a**) Time behavior of capacitance and (**b**) frequency behavior of capacitance during the adsorption of the three different gases. In all cases, pressure was maintained at 1 bar.

The characterization of the sensor against the adsorption of pure gases, such as nitrogen (N_2_), nitric oxide (NO) and difluoroethane (C_2_H_4_F_2_), was investigated. The same analysis procedure as in the case of dry air, previously presented, was followed. For the introduction of gases, the vacuum pump was connected to a Q-tube, thanks to which it was possible to insert both gases while maintaining the same pressure in the intake pipe. In all cases, gases were allowed to flow for 5 min, and then measurements were taken at regular intervals of up to 40 min after the flow was closed (Figure 8a). Then, the trend of the capacity for the gases as a function of frequency was followed (from 150 Hz to 1 kHz) (Figure 8b).

**Figure 9:**
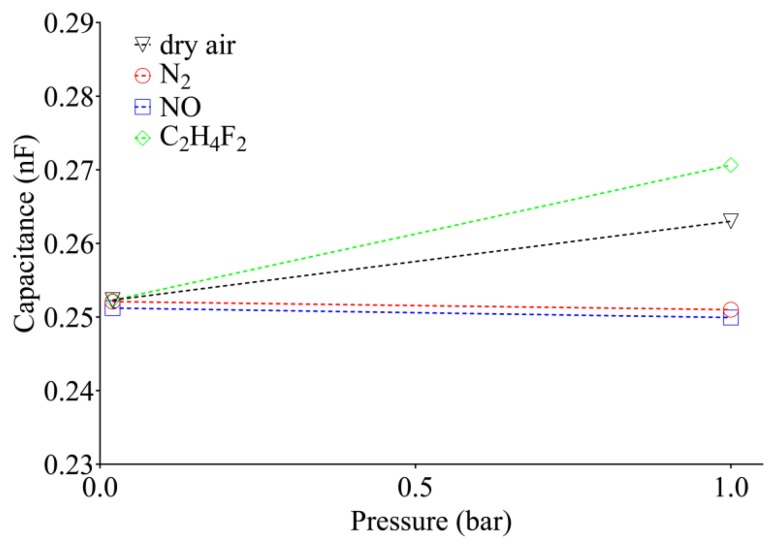
Relation between capacitance of the sample maintained at 20 mbar for 1 h and the latter after injection of gas at 1 bar for 40 min.

According to previous results obtained with dry air, Figure 8a evidences that the capacitance can be assimilated by a one-phase exponential model. In this context, the behavior of N_2_ and NO adsorption showed an exponential decay of electric capacitance with a time constant of τ_N2_ = 5.59 min and τ_NO_ = 5.43 min. According to the data previously reported for dry air, at low frequency adsorption of nitrogen, a decrease in permittivity was observed [50]. Conversely, the adsorption of C_2_H_4_F_2_ causes an increase of the electric capacitance, which can be fit with a one phase association model resulting in a higher plateau value than those found after the introduction of other gases and a higher time constant τ_C2H4F2_ = 11.65 min. It was observed that, after 60 min at 20 mbar, the capacitance is recovered to the baseline level for dry air measurements, while, after injecting N_2_, NO, and C_2_H_4_F_2_, we observed the same trend after 10 min at 20 mbar. The latter can be attributed to a residual humidity in the injected air, which requires a longer time to be removed from the framework. To get an overview of the sensor response to the different gas adsorption, Figure 9 reports the capacitance at 20 mbar and 1 bar for each gas species. 

The sensitivity was −1.3 pF/bar (N_2_), −1.3 pF/bar (NO), 10.9 pF/bar (dry air), and 18.6 pF/bar (C_2_F_4_H_2_). The capacitance variation was shown to be dependent on the kind of adsorbed gas, and the change in capacitance *C/C_0_* in the reported investigation was of +4.2% for dry air, +7.3% for C_2_H_4_F_2_, −0.4% for N_2_, and −0.5% for NO. Although the mechanism explaining the changes in electric capacitance is still not clearly explained, it is speculated that different gas molecules interact differently with the zeolite 4A composite layer, resulting in changes in the permittivity of the dielectric. This can be attributed to the different degree of polarizability of each species, which determines the adsorption affinity and thus a different capacitance effect at room temperature. The sensitivity of the zeolite-based sensor to dry air, NO N_2_, and C_2_H_4_F_2_ evidenced a high reproducibility with a relative reduction for N_2_ and NO, while a capacitance increase for dry air and C_2_H_4_F_2_ was evidenced. Moreover, relevant changes were observed after exposure to C_2_H_4_F_2_, while rather small changes were observed in case of N_2_ and NO. It is important to underline that the time necessary for the sensor to reach the plateau depends on the molecules analyzed. In fact, for air measurements, the capacity value settles at around 252 pF after about 60 min, while, after having introduced the N_2_ and NO, this value is reached only 10 min after the activation of the pump. Furthermore, if one of these gases is inserted into bell, once the vacuum pump is activated, the value of the capacity drops in a very short time to about 245 pF, and then slowly rises and settles at 252 pF. Even though the literature is wide in the development of gas sensors, lack of data was evidenced in the application of capacitive gas sensors. Ishihara et al. presented the state of the art of capacitive gas sensors for the detection of NO and CO_2_ based on metal oxides. The sensitivity is variable and generally higher than 6% (100 ppm) but the operating temperature is reported in the range 129–470 °C. Interestingly, in the proposed sensors, a capacitance reduction upon exposure to NO with respect to air is evidenced. The time response is variable from 8 to 360 s. However, in most cases, capacity did not restore to the baseline level, thus the sensors are not reusable [51]. 

Capacitive sensors for the detection of nitrogen can exploit the adsorption properties of zeolite, as in the proposed case, even though N_2_ is generally used as purge gas since it has very low affinity with the sensitive layer. Balkus et al. proposed a molecular sieve fabricated with zeolite (AlPO4-5) as a capacitive sensor for N_2_, CO, and CO_2_. The average capacitance upon exposure to nitrogen was 0.68 pF/mm^2^, while the variation with respect to water molecule adsorption was 0.21 pF/mm^2^ [52]. By considering a comparable surface area (25 mm^2^), the proposed gas sensor provides a higher C_0_ but similar capacitance variations. As previously evidenced, the zeolite-based sensors provide good sensitivity and reliable results down to room temperature. Concerning selectivity, the literature evidences zeolite is amply used as an additional layer to improve the selectivity of sensors based on other technologies (semiconductors and polymer composites), resulting in an improved selectivity [31]. The lack of selectivity remains a major limitation, even though molecular selectivity was recently discovered in zeolite (chabazite) exploiting the so-called trapdoor mechanism [30,31,32]. This mechanism allows a temperature dependent adsorption of gases by switching between standard physical adsorption and the molecular trapdoor mechanism, and is promising for the use of zeolite as a selective sensing material.

## 4. Conclusions

In the present study, the behavior of a zeolite-based capacitive sensor was investigated for the adsorption of dry air, N_2_, NO, and C_2_H_4_F_2_. The zeolite type in terms of pore diameter and structure dimension is a key factor for the optimization of adsorption capability, which results in different changes in permittivity. The dielectric characterization of samples fabricated with zeolite 4A and soybean oil mixture evidenced the possibility of manufacturing affordable gas sensors through a simple fabrication process, which avoids the use of particular chemicals and reagents. Dielectric properties are found in agreement with the data reported in the literature, and are strongly related to the type and concentration of gas species in the environment. The electric characterization evidenced the sensor is differently affected by the gas species depending on the gas molecule. The sensitivity was −1.3 pF/bar (N_2_), −1.3 pF/bar (NO), to 10.9 pF/bar (dry air) and 18.6 pF/bar (C_2_F_4_H_2_). Higher changes in capacitance were shown when the negatively charged tetrahedra of the zeolite crystal framework were exposed to dry air (C/C_0_ = +4.2%) and 1,1-difluoroethane (C/C_0_ = +7.3%), while lower dielectric variations were found upon exposure to nitrogen (C/C_0_ = −0.4%) and nitric oxide (C/C_0_ = −0.5%). The capacitance behavior versus pressure was found to be similar to a low-pressure adsorption isotherm with a low Langmuir constant. In fact, fitting of data evidenced linearity of >0.99. The proposed approach pointed out that a simple fabrication process may provide a convenient and affordable realization of reusable capacitive gas sensors.

## 5. Patents

Fiorillo, A.S. Deposition of layers of porous material layers on substrates thus obtained and devices that comprise them, ITRM20070189, 2007.

## Figures and Tables

**Table 1:** Gas characteristics.

Gas Molecule	Molecular Weight (g/mol)	Kinetic Diameter (Å)
Nitrogen (N_2_)	28.01	3.64
Nitric oxide (NO)	30.01	3.80
1,1-Difluoroethane (C_2_H_4_F_2_)	66.05	4.36 **
dry air	28.97 *	3.30–3.64

* the sum of the mole fractions of each gas multiplied by the molar mass of that particular gas; ** estimated diameter from the molecular volume [43].
